# Supplementary material for: TGFBI Facilitates Myogenesis and Limits Fibrosis in Mouse Skeletal Muscle Regeneration
Source: Int J Mol Sci. 2025 Sep 17;26(18):9042. doi: 10.3390/ijms26189042 (PMC12469635; doi:10.3390/ijms26189042)
Supplement: Supplementary file 1 [file ijms-26-09042-s001.zip › ijms-3860339-supplementary.pdf]

TGFBI Facilitates Myogenesis and Limits Fibrosis in Mouse Skeletal Muscle Regeneration

Na Rae Park [1], So-Yeon Jin [1,2], Soon-Young Kim [1], Seung-Hoon Lee [1], In-San Kim [3,4] and Jung-Eun Kim [1,2,*]

SUPPLEMENTARY MATERIALS

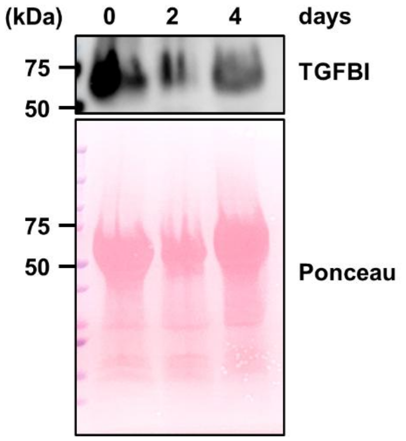

Figure S1. Western blot analysis of secreted TGFBI protein in the culture medium during C2C12 differentiation (days 0, 2, and 4). Ponceau staining was used as a loading control for protein loaded membranes during western blotting.

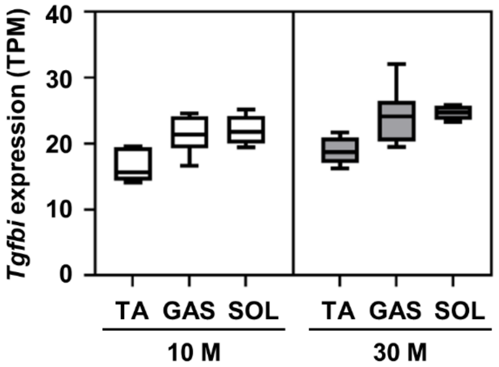

Figure S2. *Tgfbi* mRNA expression in skeletal muscles of 10- and 30-month-old mice based on SarcoAtlas (https://sarcoatlas.scicore.unibas.ch/). TA, tibialis anterior; GAS, gastrocnemius; SOL, soleus; TPM, transcripts per million; M, month.

Table S1. Antibodies used for western blotting, immunohistochemistry, and immunocytochemistry

Antibody	Catalog Number	Company
Anti-TGFBI	ab170874	Abcam (Cambridge, UK)
Anti-Myogenin	ab124800	
Anti-MYOD	sc-377460	Santa Cruz (Dallas, TX, USA)
Anti-Myosin heavy chain (MyHC)	14-6503-82	eBioscience (San Diego, CA, USA)
Goat anti-Mouse IgG (H+L) Secondary Antibody, Biotin	A16070	Invitrogen (Waltham, MA, USA)
Streptavidin, Alexa Fluor™ 488 Conjugate	S32354	
Streptavidin, Alexa Fluor™ 594 Conjugate	S32356	
Anti-α/β TUBULIN	2148S	Cell Signaling Technology (Danvers, MA, USA)
Anti-Rabbit IgG-HRP	7074S	
Anti-Mouse lgG-HRP	7076S	

Table S2. Primer sequences used for qRT-PCR

Gene		Sequence
Tgfbi	F	5'-CGG CTG AAG TCT CTC CAA GG-3'
	R	5'-TGA TGG CAT AGA CCA CAC CG-3'
Myod	F	5'-CGT GGC AGC GAG CAC TAC-3'
	R	5'-TGT AAT CCA TCA TGC CAT CAG A-3'
Myogenin	F	5'-CCC ATG GTG CCC AGT GAA-3'
	R	5'-GCA GAT TGT GGG CGT CTG TA-3'
Myh3	F	5'-CGC AGA ATC GCA AGT CAA TA-3'
	R	5'-CAG GAG GTC TTG CTC ACT CC-3'
Mymk	F	5'-GAT GGC ACT GGC CGA CTT T-3'
	R	5'-TAA CCC CAG CGG TCA TGA A-3'
Mymx	F	5'-GTG GAC CAC TCC CAG AGG AA-3'
	R	5'-TTT GAT GGG CGT TGC TGT T-3'
Tgfb1	F	5'-GCT GAC CCC CAC TGA TAC G-3'
	R	5'-CCT GTA TTC CGT CTC CTT GGT T-3'
Il1b	F	5'-GCC ACC TTT TGA CAG TGA TG-3'
	R	5'-CTT CTC CAC AGC CAC AAT GA-3'
Il6	F	5'-GGA AAT GAG AAA AGA GTT GTG C-3'
	R	5'-GTA CTC CAG AAG ACC AGA GGA-3'
Cd68	F	5'-TCC CAA CAA AAC CAA GGT CCA-3'
	R	5'-GGC TCT GAT GTA GGT CCT GTT T-3'
Cd163	F	5'-CGG CCC CAT GAA GAG GTA TC-3'
	R	5'-GAC GGT TGA CCC AGT TGT TG-3'
Pax7	F	5'-TCC ATC AAG CCA GGA GAC A-3'
	R	5'-AGG AAG AAG TCC CAC ACA G-3'
Myf5	F	5'-CAG CCC CAC CTC CAA CTG-3'
	R	5'-GCA GCA CAT GCA TTT GAT ACA TC-3'
Gapdh	F	5'-GCA TCT CCC TCA CAA TTT CCA-3'
	R	5'-GTG CAG CGA ACT TTA TTG ATG G-3'

F, forward; R, reverse
